# A clinical prediction model to identify patients at high risk of hemodynamic instability in the pediatric intensive care unit

**DOI:** 10.1186/s13054-017-1874-z

**Published:** 2017-11-20

**Authors:** Cristhian Potes, Bryan Conroy, Minnan Xu-Wilson, Christopher Newth, David Inwald, Joseph Frassica

**Affiliations:** 1 Acute Care Solutions Department, Philips Research North America, 2 Canal Park, Cambridge, MA USA; 2 Children’s Hospital Los Angeles, Los Angeles, CA USA; 3 St Mary’s Hospital, Imperial College Healthcare NHS Trust, London, UK; 4 Philips Healthcare, Andover, MA USA

**Keywords:** Hemodynamic instability, Age-dependent features, Pediatric intensive care unit, Predictive model

## Abstract

**Background:**

Early recognition and timely intervention are critical steps for the successful management of shock. The objective of this study was to develop a model to predict requirement for hemodynamic intervention in the pediatric intensive care unit (PICU); thus, clinicians can direct their care to patients likely to benefit from interventions to prevent further deterioration.

**Methods:**

The model proposed in this study was trained on a retrospective cohort of all patients admitted to a tertiary PICU at a single center in the United States, and validated on another retrospective cohort of all patients admitted to the PICU at a single center in the United Kingdom. The PICU clinical information system database (Intellivue Clinical Information Portfolio, Philips, UK) was interrogated to collect physiological and laboratory data. The model was trained using a variant of AdaBoost, which learned a set of low-dimensional classifiers, each of which was age adjusted.

**Results:**

A total of 7052 patients admitted to the US PICU was used for training the model, and a total of 970 patients admitted to the UK PICU was used for validation. On the training/validation datasets, the model showed better prediction of hemodynamic intervention (area under the receiver operating characteristic (AUROC) = 0.81/0.81) than systolic blood pressure-based (AUCROC = 0.58/0.67) or shock index-based (AUCROC = 0.63/0.65) models. Both of these models were age adjusted using the same classifier.

**Conclusions:**

The proposed model reliably predicted the need for hemodynamic intervention in PICU patients and provides better classification performance when compared to systolic blood pressure-based or shock index-based models alone. This model could readily be built into a clinical information system to identify patients at risk of hemodynamic instability.

**Electronic supplementary material:**

The online version of this article (doi:10.1186/s13054-017-1874-z) contains supplementary material, which is available to authorized users.

## Background

Early recognition and treatment of shock have been shown to decrease mortality in children in a variety of clinical scenarios [1]. However, diagnosis of shock in children, which is based on integration of patient history, vital signs, physical examination, and laboratory data, can be difficult. One challenge comes from the fact that normal-range values of clinical measurements including blood pressure, heart rate, and respiratory rate vary as children grow and age, and therefore need to be age adjusted and integrated if the clinician wants to use these measurements as early indicators of clinical deterioration [2, 3]. In addition, commonly used measures for instability such as blood pressure are often maintained within a normal range by very effective physiologic compensatory mechanisms in children until very late in the process of hemodynamic deterioration, after which point deterioration accelerates very rapidly [4].

Early warning scores are clinical decision support systems designed for initial assessment and identification of patients at risk. These systems were initially developed for use in adults, although there has been some recent research in children; the purpose of these systems is to identify patients at risk of deterioration in inpatient ward areas rather than in the ICU [5–7]. Early risk stratification using biomarkers is another promising method to identify patients at higher risk for morbidity and mortality, who might be candidates for more aggressive interventions or for clinical trial enrolment [8], but this technology is still in its infancy as immediate point-of-care testing is not possible.

The aim of this study was to construct and validate a systematic model for the early prediction of need for hemodynamic intervention (i.e., hemodynamic instability) in children already in the pediatric intensive care unit (PICU). This was defined as a clinical intervention (i.e., bolus fluid administration > 10 ml/kg/hour and/or initiation of vasoactive drugs) given to improve cardiovascular status.

## Methods

A dataset from patients admitted to the PICU in a single hospital in the United States was used for training, cross-validation, and testing of the model. Once the model had been developed, the performance of the model was validated using another dataset from a PICU in the United Kingdom.

**Fig. 1:**
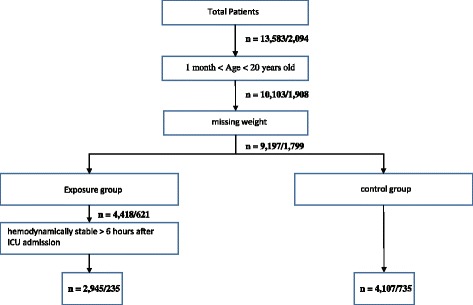
Flow diagram for exclusion criteria of encounter in the training/validation datasets. ICU intensive care unit

The dataset used for training and testing of the model was obtained retrospectively from an electronic flow sheet (Philips CareVue, Waltham, MA, USA) and a demographic database (Microsoft Access, Redmond, WA, USA) maintained by the pediatric intensivists. This dataset included records from 13,583/17,598 patients/encounters admitted to the PICU at Children's Hospital Los Angeles (CHLA) from 2003 to 2011. A patient might have been admitted several times to the PICU, thus generating more than one encounter per patient. However, only the first admission for each patient was included in the analysis. After excluding patients who did not meet the inclusion criteria shown in Fig. 1, the dataset resulted in 7052 patients.

The dataset used for the validation was obtained retrospectively from a Philips Intellispace Critical Care and Anaesthesia (ICCA) system. This dataset included records from 2094/2435 patients/encounters admitted to the PICU at St Mary’s Hospital, London, UK from 2009 to 2015. Only the first admission for each patient was included in the analysis. After excluding patients who did not meet the inclusion criteria shown in Fig. 1, the dataset resulted in 970 patients.

St Mary’s dataset was exported as a Service Evaluation which was approved by Imperial College Healthcare NHS Trust—need for individual consent was waived. IRB approval was obtained for CHLA—need for individual consent was waived. All records were completely de-identified for this study.

Both datasets originated from a nonannotated database; thus, no gold standard of hemodynamic instability was available. Instead, certain interventions by clinicians were used to demarcate hemodynamic instability events. The criteria for instability were developed based on a strong consensus among a group of experienced intensive care physicians. Each encounter was labeled as either hemodynamically unstable (i.e., exposure group) or hemodynamically stable (i.e., control group).

An encounter was labeled as hemodynamically unstable if the patient received a hemodynamic intervention. This included resuscitation with fluids (i.e., administration of a bolus fluid (colloid or crystalloid) > 10 ml/kg/hour) or initiation of vasoactive medications (i.e., dopamine, dobutamine, epinephrine, norepinephrine, neosynephrine, or vasopressin). The onset of hemodynamic intervention was defined as the time of the first intervention (either bolus fluid or vasoactive medications). Patients who were hemodynamically unstable during the first 6 hours of admission to the PICU were excluded from the analysis. A patient was labeled as hemodynamically stable if the patient did not receive any of the mentioned clinical interventions during the entire PICU stay. Only one clinical intervention was considered per patient. Each patient in the training or validation dataset was associated with a set of 36 variables (features) including vital signs, laboratory values, and ventilator parameters. Each of these features was extracted within a 24-hour observation window that preceded the hemodynamic intervention. The last measurement within the observation window was used as the value for that feature. If no measurements were found within the observation window for a specific feature, that feature was assigned a missing value (i.e., Not a Number (NaN)). A similar procedure was followed for the control group. Since there was no hemodynamic intervention in this group, variables were extracted within a random observation window during the patient stay. A patient may have invasive (i.e., arterial line) and noninvasive (i.e., blood pressure cuff) blood pressure measurements taken very close together (i.e., within 10–20 minutes difference). In this case, the noninvasive blood pressure measurement was excluded from the analysis and the invasive blood pressure measurement was used instead. Composite features were derived from a combination of other variables. The shock index was derived from heart rate (HR) and systolic blood pressure (SBP):

$$ \mathrm{Shock}\  \mathrm{index}=\frac{\mathrm{HR}}{\mathrm{SBP}.} $$

The oxygenation index (OI) and oxygen saturation index (OSI) were derived from mean airway pressure (MAP), FiO_2_, and PaO_2_ or SpO_2_:

$$ \mathrm{OI}=\frac{{\mathrm{MAP}}^{\ast }{{\mathrm{FiO}}_2}^{\ast }100}{{\mathrm{PaO}}_2}, $$

$$ \mathrm{OSI}=\frac{{\mathrm{MAP}}^{\ast }{{\mathrm{FiO}}_2}^{\ast }100}{{\mathrm{SpO}}_2}. $$

Table 1 presents all variables (input to the model) categorized by panel and component tests. All variables passed through a plausibility filter to check whether their values were in a physiologically valid range; that is, values outside the physiological range were assigned a missing value. During training, the classifier (AdaBoost) learned age-dependent risk thresholds for each variable and compensated for variability in the normal range of feature values over different age groups. Data extracted at the onset of hemodynamic intervention were used for training of the classifier. This dataset was split it into 90% for training and cross-validation, and 10% for testing.

**Table 1:** Features (units of measurement and percentage of patients with that feature recorded) for training (and validation) datasets.

Arterial blood gas			Invasive vitals		
**Arterial pH**		63 (92)	**Invasive mean blood pressure (iMBP)**	mmHg	51 (1)
Bicarbonate (HCO_3_)	mEq	63 (84)	**Invasive systolic blood pressure (iSBP)**	mmHg	51 (1)
Arterial PaCO_2_	mmHg	63 (92)	Invasive diastolic blood pressure (iDBP)	mmHg	51 (1)
SaO_2_	%	63 (79)			
**Arterial base excess (aBE)**	mEq/L	63 (91)			
**Arterial PaO**_**2**_	mmHg	63 (92)			
Ventilator parameters			Noninvasive vitals/demographics		
PF ratio		41 (26)	**Noninvasive mean blood pressure (nMBP)**	qmmHg	98 (100)
**FiO**_**2**_	%	76 (89)	**Noninvasive systolic blood pressure (nSBP)**	qmmHg	98 (100)
**Mean airway pressure**	cmH_2_O	35 (66)	Noninvasive diastolic blood pressure (nDBP)	mmHg98(100)	
			**Heart rate (HR)**	bpm	100 (100)
			**Respiratory rate (RR)**	bpm	99 (96)
			**SpO**_**2**_	%	61 (68)
			**Age**	years	100 (100)
			**Temperature (T)**	Celsius	99 (99)
Basic metabolic panel			Comprehensive metabolic panel		
**Glucose**	mg/dl	74 (91)	Alanine aminotransferase (ALT)	U/L	18 (77)
Chloride	mEq/L	72 (91)	**Albumin**	g/dl	18 (81)
**Blood urea nitrogen (BUN)**	mg/dl	66 (84)	Total protein	g/dl	18 (81)
Creatinine	mg/dl	66 (84)			
Potassium	mEq/L	78 (91)			
Sodium	mEq/L	77 (93)			
Complete blood count					
WBC – leukocytes	K/μl	65 (43)	**RBC**	M/μl	65 (12)
**Hemoglobin**	g/dl	68 (52)	Platelets	K/μl	65 (41)
Additional tests					
Magnesium	mg/dl	26 (70)	**Prothrombin time (INR)**	seconds	37 (29)
Lactic acid	mg/dl	14 (45)	**Urine output**	cm^3^/kg/hour	77 (72)

All 36 features were input to the AdaBoost-abstain classifier to classify hemodynamic instability. Among the 36 features, only 21 (highlighted in bold) were selected by the model

*RBC* red blood cells, *WBC* white blood cells

Once the classifier was trained, the performance of the algorithm was tested and validated on variables extracted within a 24-hour observation window that preceded (i.e., 1–12 hours) the onset of hemodynamic intervention. Algorithm performance was measured by the area under the ROC (AUROC) curve at different prediction times.

The final dataset for training and testing of the model (i.e., CHLA) has 36 variables and 42% instability prevalence (2945 hemodynamically unstable, 4107 hemodynamically stable). The final dataset for validation of the algorithm (St Mary’s Hospital) has 36 variables and 24% instability prevalence (235 hemodynamically unstable, 735 hemodynamically stable). Table 2 shows summary statistics stratified by exposure (hemodynamically unstable) and control (hemodynamically stable) groups.

**Table 2:** Summary statistics stratified by exposure and control groups.

	All observations	Exposure group	Control group
	*N* = 7052 (970)	*N* = 2945 (235)	*N* = 4107 (735)
Mean age (years)	6.9 (4.3)	6.6 (3.9)	7.1 (4.4)
Mechanically ventilated (%)	39.3 (66.2)	56.1 (85.9)	28.5 (60.4)
Mean PICU LOS (days)	8.2 (6.6)	15.3 (13.5)	3.6 (4.6)
Mortality (%)	3 (4.2)	6 (14.9)	1.1 (1.1)

Values in parentheses correspond to the validation dataset

*LOS* length of stay, *PICU* pediatric intensive care unit

### Model development

The AdaBoost classifier was used for development of the model. AdaBoost is a very effective machine learning technique for constructing a powerful ensemble classifier from a weighted majority vote of simpler (“weak”) classifiers. In this work, the weak classifiers are simple decision stumps that predict the hemodynamic status (stable/unstable) based on a combination of a single feature (e.g., heart rate) and the patient’s age. Thus, each weak classifier can be thought of as a lookup table based on patient age and another feature. Including the patient’s age in each weak classifier allows us to learn age-dependent risk thresholds and compensate for variability in the normal range of feature values over age groups. For instance, a 14-year-old child with a shock index (SI) of 1.2 will be at a higher risk of hemodynamic instability than a 1 year old with the same SI value (see Additional file 1: Figure S1).

A slight variation of the AdaBoost classifier [9] (AdaBoost-abstain) was employed to handle missing features. Certain features that were highly predictive of hemodynamically instability when measured, such as lactic acid, were missing on 86% or more of the patient encounters in the training dataset. Rather than discard sporadically measured features entirely or impute missing values, AdaBoost-abstain allows each weak classifier to abstain from voting if its dependent feature is missing. Thus, weak classifiers produce one of three outputs (either hemodynamically stable, hemodynamically unstable, or no vote (abstain)). AdaBoost-abstain was trained and tested using 10 cross-validation folds. Each fold had roughly equal size and roughly the same class proportions as the whole dataset.

## Results

Thirty-six features were input into the AdaBoost-abstain classifier during training, and only 21 were selected by the classifier. Among those 21 selected features, SI, pH, mean airway pressure, and normalized urine output (ml/kg/hour) were the most discriminative predictors of the model. In the cross-validation results using the training dataset, the model predicted hemodynamic instability with an AUROC of 0.80 (0.70), sensitivity of 0.66 (0.50), specificity of 0.78 (0.77), positive predictive value (PPV) of 0.68 (0.62), negative predictive value (NPV) of 0.76 (0.68), and positive likelihood ratio (LR) of 5.22 (3.26) at 1 and 12 hours before hemodynamic intervention. Values in parentheses correspond to the performance at 12 hours before hemodynamic intervention. The optimal threshold (i.e., probability > 0.47 classifies an encounter as hemodynamically unstable) was selected such that sensitivity (recall) and precision were equal. PPV and NPV were adjusted for 42% prevalence of hemodynamic instability.

The classification performance of the algorithm in the training dataset was evaluated across five age groups (1–12 months old and 1–3, 3–6, 6–12, and 12–20 years old,) and different sets of features (vital signs, laboratory data, ventilator parameters). The classification performance across the five age groups (presented in Table 3) was very similar (mean AUROC = 0.78, standard deviation (SD) = 0.04, minimum = 0.74, maximum = 0.83). The overall performance when only vital signs or laboratory values were included in the model was AUROC of 0.71 and 0.70, respectively. Each set of features alone was a good predictor of hemodynamic instability, but the classification performance was better when vitals and laboratory values were all combined into one model (AUROC = 0.77). The impact of mechanical ventilation in the algorithm performance was also evaluated. The classification performance for patients on mechanical ventilation (AUROC = 0.78) was very close to patients who were not (AUROC = 0.77).

**Table 3:** Algorithm performance across different age groups.

Age group	*N* ^a^	AUROC^b^
1–12 months	165/300	0.82/0.78
1–3 years	98/245	0.77/0.82
3–6 years	94/147	0.82/0.72
6–12 years	151/159	0.74/0.89
12–20 years	191/105	0.75/0.85

^a^Number of patients for that particular age group (training/validation)

^b^Reported at the time of hemodynamic intervention (training/validation)

*AUROC* area under receiver operating characteristic curve

### Model validation

Once the parameters of the model were learned using the training dataset, the final model was validated using data from a different PICU (St Mary’s Hospital). The classification performance of the algorithm in the validation dataset was AUROC of 0.81 (0.74), sensitivity of 0.76 (0.66), specificity of 0.70 (0.71), PPV of 0.45 (0.42), NPV of 0.90 (0.87), and positive LR of 2.65 (2.27) at 1 and 12 hours before hemodynamic intervention. The optimal threshold (i.e., probability > 0.57 classifies an encounter as hemodynamically unstable) was selected such that sensitivity (recall) and precision were equal. PPV and NPV were adjusted for 24% prevalence of hemodynamic instability. Classification performance on the validation dataset was very similar across the five age groups (mean AUROC = 0.81, SD = 0.06, minimum = 0.72, maximum = 0.89) (see Table 3).

### Comparison against blood pressure-based or SI-based models

**Fig. 2:**
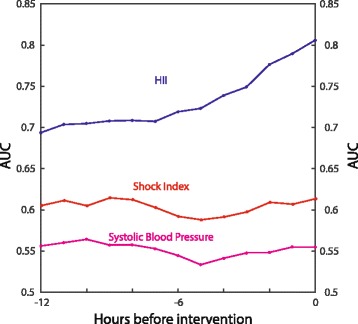
Model performance on training dataset. Using shock index (SI) alone, one can detect hemodynamic instability hours before clinical intervention, much earlier than relying on systolic blood pressure alarms (AUC = 0.63); but, using our model, which combines SI and other measurements, further improves the early detection (AUC = 0.81). *AUC* area under the curve, *HII* hemodynamic instability indicator

To test the hypothesis that the proposed model is superior to systolic blood pressure-based or SI-based models for the early detection of hemodynamic instability, the classification performance of the three models was evaluated on the training and validation datasets. Both models were adjusted by age using the same classifier. Figure 2 shows that, on average, the proposed model improves the AUROC of the systolic blood pressure-based model by almost 15%, and of the SI-based model by 11%.

### Predicting the need for vasoactive agents only

Among 2945 encounters labeled as hemodynamically unstable in the training dataset, 70% received a bolus of fluid > 10 ml/kg/hour and 30% received vasoactive medications. To evaluate whether the model was biased towards a bolus of fluid (>10 ml/kg/hour) as the first intervention, we calculated the performance of the model on encounters where the first intervention was vasoactive medication. The performance of the model was AUC = 0.88/0.87 in the training (CHLA) and validation (St Mary’s) datasets, respectively. Thus, the model performs and generalizes well on those unstable encounters where the first intervention was a vasoactive medication.

### Algorithm structure

**Fig. 3:**
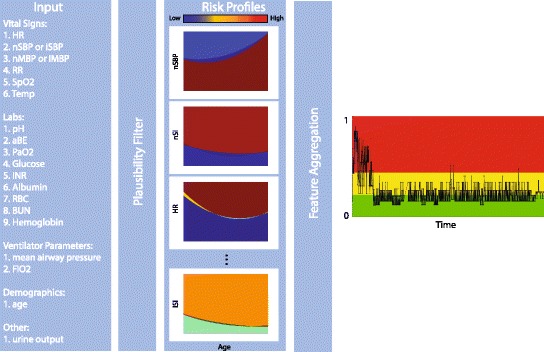
Schematic of algorithm. The algorithm receives as input 21 features from vital signs, laboratory, ventilator measurements, normalized urine output, and age. The algorithm first filters input values in valid ranges, secondly determines feature value thresholds that depend on the age of the patient, thirdly determines feature contribution prediction scores, and finally aggregates the individual feature contribution prediction scores to determine a hemodynamic instability indicator (HII). The HII is a score, on a scale from 0 to 1, representing the probability of a patient to be hemodynamically unstable. This score is mapped to three colors to indicate the risk level of deterioration (i.e., green for low risk, yellow for medium risk, and red for high risk). aBE arterial base excess, BUN blood urea nitrogen, FiO fraction of inspired oxygen, HR heart rate, iMBP invasive mean blood pressure, INR international normalized ratio, iSBP invasive systolic blood pressure, iSI invasive shock index, nMBP noninvasive mean blood pressure, nSBP noninvasive systolic blood pressure, nSI noninvasive shock index, PaO2 arterial partial pressure of oxygen, RBC red blood cells, RR Respiratory rate, SpO2 oxygen saturation

The model was implemented in ICCA Rel H as one of the algorithms included in the clinical decision support flowsheet. The algorithm receives as input 21 features: vital signs (heart rate, noninvasive or invasive systolic blood pressure, noninvasive or invasive mean blood pressure, respiration rate, SpO_2_, temperature), laboratory measurements (pH, PaO_2_, aBE, INR, albumin, glucose, hemoglobin, RBC, BUN), ventilator measurements (mean airway pressure, FiO_2_), normalized urine output, and age (Fig. 3). These measurements are first filtered allowing only reasonable data (i.e., measurements in a physiologically valid range) to be passed for further processing. The algorithm expects new measurements added as they become available in a time-sequential manner. At a minimum, heart rate and age are required for the algorithm to produce an output. For frequent output of the algorithm, it is suggested that heart rate and blood pressure are made available to the algorithm via an automated interface as frequent as once a minute. However, if vital signs are supplied at a lower frequency, such as hourly with nurse charting, the algorithm will still output a score that is predictive of hemodynamic instability. The minimum frequency for laboratory, blood pressure, and ventilator measurements is 24 and 1 hour, respectively. If no measurements are made in the last 24 hours (for laboratory measurements) or 1 hour (for blood pressure measurements), the measurement is considered missing.

Once the feature values are filtered, the algorithm determines feature value thresholds that depend on the age of the patient, then determines feature contribution prediction scores, and finally aggregates the individual feature contribution prediction scores to determine a hemodynamic instability indicator (HII). The HII is a score, on a scale from 0 to 1, that represents the probability of a patient to be hemodynamically unstable (i.e., to receive a hemodynamic intervention with fluid bolus or cardiovascular medications). This score is mapped to three colors to indicate the risk level of deterioration (i.e., green for low risk, yellow for medium risk, and red for high risk as shown in Fig. 3).

## Discussion

Our proposed model can be used to identify patients at risk of hemodynamic instability in the hours before the clinical intervention and provides better classification performance results compared to systolic blood pressure-based or SI-based models. The algorithm has the advantage of handling missing values without doing imputation, of adjusting features that are age dependent, and of integrating different sources of clinical data to produce a single risk score that the caregiver can easily interpret. The algorithm outputs a risk score (i.e., probability of clinical deterioration) that could be used to intelligently allocate clinical resources and identifies high-risk patients.

The shock index may predict outcomes of adults in intensive care [10]. However, its adoption in children is limited since an age-standardized SI is difficult to assess, especially in the context of chronic illness [11]. The hemodynamic instability risk profiles proposed in our model may be a solution to this problem since features such as SI are age-standardized using a purely data-driven approach (see Additional file 1: Figure S1). Our model includes clinical features that are in line with current understanding of the clinical manifestations of early shock. It is known that during the early stages of circulatory shock the body activates mechanisms to compensate for the lack of oxygen delivery to the main organs and tissues [1]. Mainly, the heart rate increases by activation of the sympathetic nervous system, and urine output decreases by activation of the renin–angiotensin–aldosterone system. Other clinical features such as cold extremities, mottled skin, and prolonged capillary refill time were not considered in the model as they are signs of decompensated shock and thus represent the physiological state clinicians are trying to avoid [12].

Our model learned to associate an increased risk of hemodynamic instability during any time that a patient was invasively monitored (i.e., placement of an arterial line) or was on mechanical ventilation. However, the classification performance was not significantly reduced when invasive measurements or ventilator parameters were considered missing in the model (a 2% drop in classification performance). Although the final model was mainly trained for predicting hemodynamic interventions (i.e., fluid bolus or initiation of vasoactive drugs), the same model could be used for tracking the patient’s response to therapy. Further research and prospective studies are needed to answer this question.

### Data limitations and challenges

One of the major challenges and most time-consuming task in the development of prediction models has concerned the use of nonstandard terminology within individual hospital information systems [13]. Most hospital and laboratory information systems’ catalogs have very similar data under diverse naming schemas. Time and effort were required to understand the data and coding of the electronic data elements recorded in the individual institutional databases in this study. Cases were found with different clinical event names, different clinical event codes, but similar data distribution; or cases were found with similar clinical event names, different clinical event codes, but different data distribution. After a long data cleaning process and discussion with pediatric critical care experts and IT personnel at each institution, it was possible to map distinct clinical event codes referring to the same data.

Another limitation in the development of the model was the difference in the patient population between the two PICUs. For instance, patients in the US PICU had more arterial lines placed (51%) than patients in the UK PICU (12%), but more patients in the UK PICU were mechanically ventilated (66%) when compared to patients in the US PICU (39%). Ideally, one would like to train a model from several PICUs located across different geographical regions and different levels of care with the hope that the model will learn parameters that are generalizable when testing on new data coming from other PICUs. However, this approach is not always feasible, and most clinical decision support algorithms found in the literature are trained and tested from a single hospital, but rarely validated across other hospitals. However, the fact that the score functioned well in two different environments with two heterogeneous populations could also be seen as a strength. Indeed, our model generalized well when validated on the UK PICU (AUROC = 0.81) without extra tuning of the model parameters. Nevertheless, further validation of our model using data collected from PICUs across different hospitals in the United States and other countries is needed.

Another disadvantage of labeling hemodynamic instability events based on clinical interventions is that interventions often occur before the patient was transferred to the PICU [14]. As this information was not available in our databases, the issue was mitigated by including in the analysis only patients who were stable for at least 6 hours after being admitted to the PICU.

### Future work

The model presented here was trained and validated on retrospective data from two different hospitals. Although the results are promising, prospective validation work is also required to confirm our findings. The goals of a prospective study would be to determine the clinical value of running the algorithm and displaying the risk score to clinicians in the ICU. Clinical outcomes of such a study might include time to hemodynamic interventions, ventilator-free days, length of stay, and mortality. An important part of study design would be the integration of such predictive algorithms into the clinical workflow, without which clinical outcomes would not be improved.

## Conclusion

This retrospective cohort study demonstrated that our proposed prediction model derived from vital signs, ventilator parameters, and laboratory values can be used to early identify patients at greater risk of hemodynamic instability. The validation results indicate that the use of our prediction model instead of an assessment based on blood pressure or shock index measurements alone improves the early detection of patients at higher risk of hemodynamic instability.

## Additional file

Additional file 1: is Figure S1 showing hemodynamic instability risk profile for shock index (SI). A 14-year-old child with SI = 1.2 will be at a higher risk of hemodynamic instability than a 1 year old with the same SI value (EPS 1840 kb)

## Abbreviations

AUCROC: Area under an ROC curve
CHLA: Children’s Hospital Los Angeles
HII: Hemodynamic instability indicator
ICCA: Philips Intellispace Critical Care and Anaesthesia
LR: Likelihood ratio
NPV: Negative predictive value
PICU: Pediatric intensive care unit
PPV: Positive predictive value
SD: Standard deviation
SI: Shock index
